# Prognostic comparison between superior and basal segments in pure-solid non-small cell lung cancer

**DOI:** 10.1007/s11748-025-02202-6

**Published:** 2025-09-18

**Authors:** Shinji Yuhara, Masaaki Nagano, Yue Cong, Keita Nakao, Mitsuaki Kawashima, Gouji Toyokawa, Chihiro Konoeda, Yan Luo, Masaaki Sato

**Affiliations:** 1 Department of Thoracic Surgery, The University of Tokyo Graduate School of Medicine, 7-3-1, Hongo Bunkyo-ku, Tokyo, Japan; 2 Center for Medical Education and Internationalization, Kyoto University Graduate School of Medicine, Yoshida-Honmachi, Sakyo-ku, Kyoto-shi, Kyoto, Japan

**Keywords:** Lung cancer, Pure-solid, Superior segment, Lobectomy, Prognosis

## Abstract

**Objective:**

Studies suggest that non-small cell lung cancer of the superior segment (S6) affects different lymphatic pathways and results in worse prognosis than basal segment tumors. We aimed to compare survival after lobectomy between non-small cell lung cancer in the S6 and basal segments, focusing specifically on pure-solid tumors, which have higher lymph node metastasis rates and worse prognosis.

**Methods:**

We retrospectively reviewed patients with pure-solid, clinical N0 M0, ≤ 5-cm, lower-lobe non-small cell lung cancer who underwent lobectomy with hilar and mediastinal lymphadenectomy between April 2009 and December 2021. Overall survival, recurrence-free survival and clinicopathological characteristics were evaluated.

**Results:**

We categorized 157 patients into S6 (n = 58) and basal segment (n = 99) groups. The 5-year overall survival (66.4% vs. 68.6%, respectively; p = 0.519; hazard ratio, 1.19; 95% confidence interval, 0.70–2.03), and recurrence-free survival (54.8% vs. 65.5%, respectively; p = 0.452; hazard ratio, 1.22; 95% confidence interval, 0.72–2.06) rates were comparable between the S6 and basal segment groups. Multivariable Cox regression analyses indicated that tumor location was not associated with overall or recurrence-free survival. The S6 group showed a higher tendency for visceral pleural invasion compared with the basal segment group. Superior mediastinal lymph node metastasis was pathologically confirmed exclusively in the S6 group (two cases).

**Conclusions:**

No significant difference in survival was observed between S6 and basal segment pure-solid non-small cell lung cancer after lobectomy with hilar and mediastinal lymph node dissection.

**Supplementary Information:**

The online version contains supplementary material available at 10.1007/s11748-025-02202-6.

## Introduction

Previous studies have highlighted differences in clinicopathological characteristics and prognosis between non-small cell lung cancer (NSCLC) originating in the superior segment (S6) and basal segment following radical resection [1–4]. A retrospective study on solid-dominant lower lobe lung cancers suggested that S6 tumor location is a poor prognostic factor [1], while another study reported comparable prognoses between S6 and basal segment NSCLC following lobectomy [2]. Recently, interest in S6 lung cancers has increased [1, 4–8], with studies examining distinct lymph node metastatic patterns that differ from those of other lung segments. Watanabe et al. analyzed lower-lobe N2 tumors and found a significantly higher rate of superior mediastinal metastasis in S6 tumors compared to basal segment tumors [4]. Pure-solid NSCLC, compared to lung cancers with ground-glass opacity (GGO) components, is associated with a higher incidence of lymph node metastasis (LNM) and worse prognosis [9–11]. Consequently, it was hypothesized that focusing exclusively on pure-solid NSCLC might reveal prognostic differences after radical resection between S6 and basal segment tumors. While previous studies have examined S6 and basal segment lung cancers, particularly in cases involving GGO components, research on pure-solid NSCLC remains limited. This study aimed to investigate the clinicopathological and prognostic differences between S6 and basal segment pure-solid NSCLC through retrospective review and multivariable analysis. Patients with tumors larger than 5 cm were excluded owing to the difficulty in determining tumor origin.

## Subjects

### Ethical statement

This retrospective study was approved by the Ethics Committee of the University of Tokyo Hospital (Approval No. 2406-9) in March 2024 and conducted in accordance with the principles of the Declaration of Helsinki. Owing to its retrospective observational design, the requirement for informed consent was waived.

### Patients

Patients with primary lower lobe lung cancer who underwent lobectomy at the University of Tokyo Hospital between April 2009 and December 2021 were retrospectively reviewed. The study included those with pure-solid NSCLC ≤ 5 cm, classified as clinical node-negative metastasis-negative (cN0M0), who underwent lobectomy with hilar and mediastinal lymph node dissection (LND). Exclusion criteria included patients with preoperatively diagnosed LNM, distant metastasis, tumors containing GGO components, small cell carcinoma, and tumors > 5 cm in diameter owing to challenges in determining tumor origin. Additionally, patients who underwent lobectomy without mediastinal lymphadenectomy were excluded due to inadequate pathological staging. Tumor location was determined based on preoperative chest computed tomography (CT) findings, with the V6b and V6c branches of the pulmonary vein serving as the boundary between groups. Tumors involving both segments were classified according to the segment containing the larger component of the tumor.

Collected patient data included age, sex, smoking history, respiratory function, tumor side and segment, radiological tumor size, preoperative carcinoembryonic antigen (CEA) levels, lymphadenectomy extent (selective or systematic), tumor histology (adenocarcinoma, squamous cell carcinoma, or others), pathological N factor, pathological stage, lymphatic invasion, venous invasion (v0 or v1–2), visceral pleural invasion (VPI) (pl0 or pl1–3), pulmonary metastasis (pm0 or pm1), maximum standardized uptake value (SUVmax) derived from fluorodeoxyglucose-positron emission tomography (FDG-PET), and adjuvant and neoadjuvant therapy. In this study, sex was defined as a set of biological characteristics. Selective LND was defined as the dissection of the lower mediastinal nodes only, whereas systematic LND included the dissection of both the lower and upper mediastinal nodes. Tumor-Node-Metastasis (TNM) staging followed the eighth edition of the TNM Classification of Malignant Tumors, and histological classification was based on the World Health Organization criteria [12]. For the 95 cases prior to December 2016, pathological invasive size was not recorded; therefore, the maximum tumor diameter was used as a surrogate to determine the pathological stage. Because this study included only pure-solid tumors, the difference between maximum diameter and invasive size was considered negligible in terms of its impact on staging.

### Patient follow-up

The day of surgery was designated as time zero. Postoperative monitoring included chest CT every 6 months for a minimum of 5 years. When recurrence was suspected based on CT findings or symptoms, further assessments were conducted using FDG-PET and brain magnetic resonance imaging.

## Methods

### Outcome evaluation

The primary objective was to compare overall survival (OS) between the S6 and basal segment groups. OS was defined as the interval from the date of surgery to death from any cause or the most recent follow-up. Secondary objectives included evaluating differences in recurrence-free survival (RFS) and clinicopathological characteristics between the 2 groups. RFS was defined as the period from the date of surgery to the first recurrence, death, or the last recorded follow-up.

### Statistical analysis

Categorical variables are expressed as numbers and percentages, while continuous variables are presented as means with standard deviation ( ± ) or medians with interquartile ranges for skewed distributions. Survival data were visualized using the Kaplan–Meier method, with survival differences between groups tested by the log-rank test. Multivariable Cox regression analyses were performed to evaluate whether tumor location (S6 or the basal segment) was a prognostic factor for OS and RFS. Based on previous studies [12–16], sex, age, tumor size, the presence of pathologically confirmed LNM, and VPI were adopted as covariates. Statistical analyses were conducted using EZR version 1.36 (Saitama Medical Center, Jichi Medical University, Saitama, Japan), a graphical user interface for R (The R Foundation for Statistical Computing, Vienna, Austria) [17]. A *P*-value < 0.05 was considered statistically significant.

### Subgroup analysis

Given the considerable evidence highlighting the significance of sublobar resection for tumors measuring 2 cm or smaller [18], we conducted exploratory subgroup analyses of S6 versus basal segment tumors in patients with tumors ≤ 2 cm and in those with tumors > 2 cm.

## Results

### Patient characteristics and pathological outcomes

**Fig. 1:**
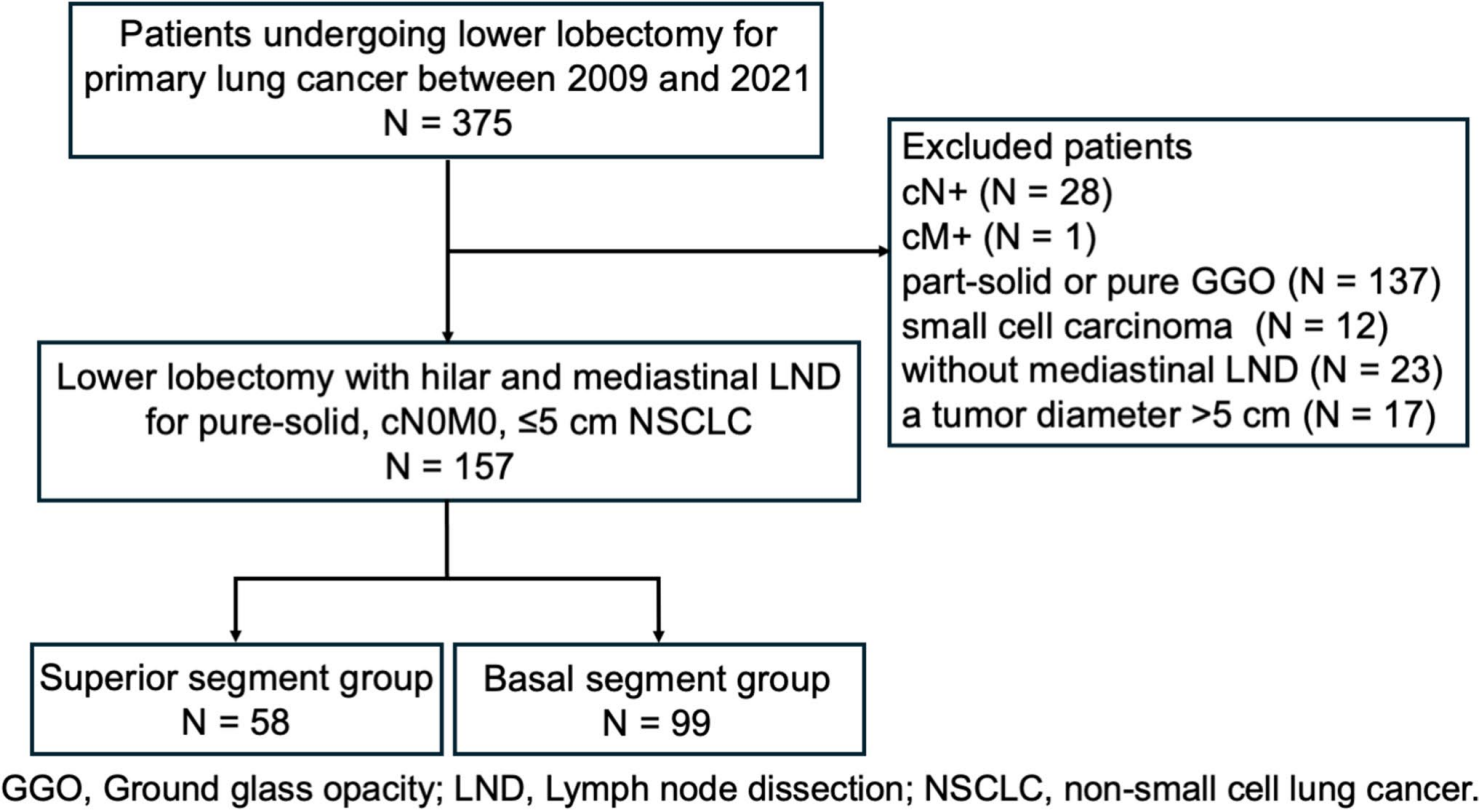
Patient inclusion criteria

Overall, 375 patients underwent lobectomy for primary lower lobe lung cancer between April 2009 and December 2021. Exclusion criteria included preoperatively diagnosed LNM (n = 28), distant metastasis (n = 1), GGO components (n = 137), small cell carcinoma (n = 12), or tumors > 5 cm in diameter (n = 17). Additionally, patients who underwent lobectomy without mediastinal LND (n = 23) were excluded. Ultimately, 157 patients with pure-solid, cN0M0 NSCLC ≤ 5 cm in diameter who underwent lobectomy with hilar and mediastinal LND were included. These patients were divided into two groups: S6 (n = 58) and basal segment (n = 99) (Fig. 1). Patient characteristics are summarized in Table 1. SUVmax data was missing for a relatively large proportion of patients: 11 patients (19.0%) in the S6 group and 27 patients (27.3%) in the basal segment group. No patients underwent neoadjuvant therapy. The basal segment group had significantly higher levels of preoperative serum CEA compared to the S6 group (*P* = 0.04), whereas SUVmax was significantly higher in the S6 group (*P* = 0.04). Other factors showed no significant differences. Pathological outcomes are described in Table 2. The incidence of VPI was significantly higher in the S6 group, affecting 28 of 58 patients (48.3%) compared to 28 of 99 patients (28.3%) in the basal segment group (*P* = 0.02). However, other variables, including tumor histology, pathologically confirmed LNM, lymphatic invasion, vascular invasion, and pulmonary metastasis, did not differ substantially between the two groups. Systematic mediastinal LND was performed in 16 patients (27.6%) in the S6 group and 29 patients (29.3%) in the basal segment group. Among those undergoing systematic lymphadenectomy, superior mediastinal LNM was pathologically confirmed in two cases (12.5%) in the S6 group but was not observed in the basal segment group.

**Table 1:** Patient characteristics in S6 and basal segment groups

Variables	S6 (N = 58)	Basal segment (N = 99)	*P*-value^a^
Age (years), mean ± SD	71.9 ± 8.5	70.2 ± 10.6	0.30
Sex, n (%)			
Male	40 (69.0)	67 (67.7)	1.00
Female	18 (31.0)	32 (32.3)	
Brinkman Index, mean ± SD	738 ± 689	726 ± 691	0.91
%VC (%), mean ± SD	103 ± 16	102 ± 15	0.63
FEV1 (mL), mean ± SD	2202 ± 708	2192 ± 648	0.92
FEV1% (%), mean ± SD	70.5 ± 9.9	71.3 ± 9.9	0.64
Laterality, n (%)			
Left	29 (50.0)	48 (48.5)	0.89
Right	29 (50.0)	51 (51.5)	
Radiological tumor size (mm), mean ± SD	26.7 ± 10.9	25.3 ± 10.1	0.40
CEA (ng/mL), median (IQR)	3.9 (2.6–5.8)	5.1 (3.1–7.8)	0.04
SUVmax, median (IQR)	5.1 (3.4–10.8)	4.4 (2.7–7.2)	0.04

*CEA* carcinoembryonic antigen, *FEV1* forced expiratory volume in one second, *FEV1%* forced expiratory volume in one second (expressed as forced vital capacity ratio), *IQR* interquartile range, *%VC* percentage of predicted vital capacity, *SD* standard deviation, SUVmax maximum standardized uptake value, *S6* superior segment

^a^*P*-values were not adjusted for multiple testing and should be interpreted with caution

**Table 2:** Pathological and postoperative characteristics in S6 and basal segment groups

Variables	S6 (N = 58)	Basal segment (N = 99)	*P*-value^a^
Mediastinal LND, n (%)			
Selective	42 (72.4)	70 (70.7)	0.86
Systematic	16 (27.6)	29 (29.3)	
Histology, n (%)			
Adenocarcinoma	37 (63.8)	64 (64.7)	0.99
Squamous cell carcinoma	16 (27.6)	27 (27.3)	
Others	5 (8.6)	8 (8.1)	
Pathological N factor, n (%)			
N0	46 (79.3)	76 (76.8)	0.65
N1	6 (10.4)	15 (15.2)	
N2	6 (10.4)	8 (8.1)	
Detail of mediastinal LNM, n (%)			
Superior	2 (3.5)	0 (0)	0.26
Inferior	5 (8.6)	10 (10.1)	0.98
Pathological stage, n (%)			
IA1	3 (5.2)	7 (7.1)	0.33
IA2	9 (15.5)	15 (15.2)	
IA3	7 (12.1)	18 (18.2)	
IB	20 (34.5)	24 (24.2)	
IIA	0 (0)	8 (8.1)	
IIB	9 (15.5)	13 (13.1)	
IIIA	8 (13.8)	10 (10.1)	
IIIB	2 (3.4)	2 (2.0)	
IVA	0 (0)	2 (2.0)	
Lymphatic invasion, n (%)	18 (31.0)	34 (34.3)	0.80
Venous invasion, n (%)	35 (60.4)	46 (46.5)	0.13
Visceral pleural invasion, n (%)	28 (48.3)	28 (28.3)	0.02
Pulmonary metastasis, n (%)	5 (8.6)	3 (3.0)	0.25
Adjuvant therapy, N (%)	15 (26.3)	15 (15.2)	0.14

*S6* superior segment, *LND* lymph node dissection, *LNM* lymph node metastasis

^a^*P*-values were not adjusted for multiple testing and should be interpreted with caution

### Survival analysis

**Fig. 2:**
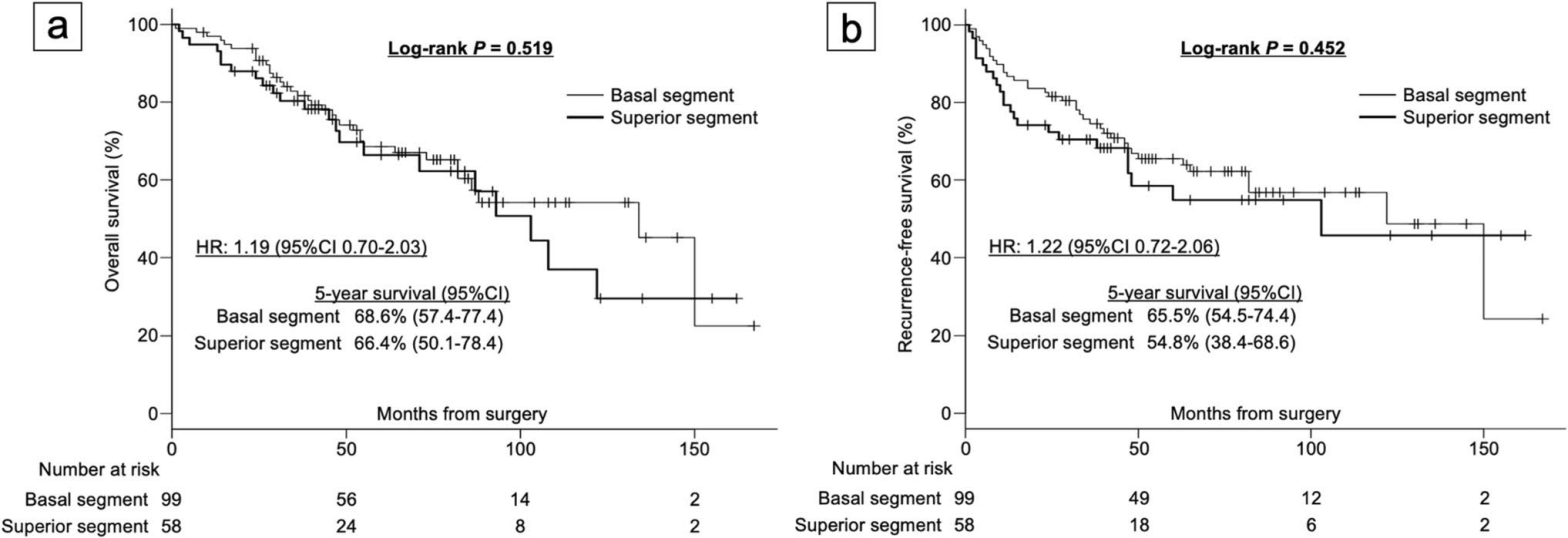
Survival curves for 2 groups. **a** Overall survival curves for the superior segment (thick line) and basal segment groups (thin line). **b** Recurrence-free survival curves for the superior segment (thick line) and basal segment groups (thin line)

The median follow-up time was 52 (range 0–167) months, with 43.5 (range 2–162) months in the S6 group and 54 (range 0–167) months in the basal segment group. The 5-year OS probability was 66.4% (95% confidence interval [CI] 50.1%–78.4%) in the S6 group and 68.6% (95% CI 57.4%–77.4%) in the basal segment group. No significant difference in OS was observed between the groups (log-rank *P* = 0.519; hazard ratio [HR]: 1.19; 95% CI 0.70–2.03) (Fig. 2a). Similarly, the 5-year RFS probability was 54.8% (95% CI 38.4%–68.6%) in the S6 group and 65.5% (95% CI 54.5%–74.4%) in the basal segment group. No significant difference in RFS was found between the groups (log-rank *P* = 0.452; HR 1.22; 95% CI 0.72–2.06) (Fig. 2b).

### Multivariable analysis

Multivariable Cox regression analyses were performed to assess whether tumor location was a prognostic factor for OS and RFS (Table 3), adjusting for age, sex, tumor size, pathological N status, and VPI. Tumor location was not identified as a significant prognostic factor for OS (HR, 1.15; 95% CI, 0.67–1.98; *P* = 0.60) or RFS (HR 1.12; 95% CI, 0.66–1.90; *P* = 0.67). Thus, OS or RFS were not significantly associated with tumor location.

**Table 3:** Multivariable Cox regression analysis for overall and recurrence-free survival

Variables	HR	95% CI	*P*-value
Overall survival			
Tumor location, superior vs. basal	1.06	0.61–1.84	0.84
Age (years)	1.05	1.01–1.09	0.02
Sex, male vs. female	1.04	0.57–1.90	0.90
Radiological tumor size (mm)	1.02	1.00–1.05	0.12
Pathological N status, N0 vs. N1–2	4.14	2.37–7.24	< 0.001
Visceral pleural invasion, pl0 vs pl1–3	1.61	0.93–2.80	0.09
Recurrence-free survival			
Tumor location, superior vs. basal	1.02	0.60–1.74	0.95
Age (years)	1.04	1.00–1.08	0.03
Sex, male vs. female	1.14	0.63–2.06	0.66
Radiological tumor size (mm)	1.01	0.99–1.04	0.39
Pathological N status, N0 vs. N1–2	3.78	2.17–6.58	< 0.001
Visceral pleural invasion, pl0 vs pl1–3	1.91	1.11–3.28	0.02

*CI* confidence interval, *HR* hazard ratio, *S6* superior segment

### Subgroup analysis

Patient characteristics and pathological outcomes for the S6 group versus the basal segment group among patients with tumors ≤ 2 cm are presented in Supplementary Tables 1 and 2, whereas those for patients with tumors > 2 cm are shown in Supplementary Tables 3 and 4. In the subgroup with tumors ≤ 2 cm as well as in the subgroup with tumors > 2 cm, no significant differences in OS or RFS were observed between the S6 and basal segment groups (Supplementary Figs. 1 and 2).

## Discussion

No significant difference in prognosis was observed between the S6 and basal segment groups when lobectomy with hilar and mediastinal LND was performed for pure-solid, ≤ 5 cm, cN0M0 NSCLC originating in the lower lobe. Regarding pathological features, the incidence of VPI was higher in the S6 group compared to the basal segment group. Although the incidence of pathologically confirmed LNM did not differ markedly between the 2 groups, superior mediastinal LNM was observed in two cases in the S6 group but not in the basal segment group.

For pure-solid, ≤ 5 cm, cN0M0, lower-lobe NSCLC, the findings revealed no significant difference in prognosis between the two groups when lobectomy was performed along with hilar and mediastinal lymphadenectomy. This finding was consistent across tumor-size subgroups, with no significant differences in OS or RFS between the S6 and basal segment groups. However, as this was a post-hoc subgroup analysis, the result should be confirmed in future studies. Several studies have compared the prognosis of S6 versus basal segment lung cancer [1–3, 5]. Handa et al. retrospectively analyzed cases of clinical stage I solid-dominant NSCLC that underwent lobectomy or segmentectomy with systematic LND, classifying them into S6 and basal segment groups [1]. Their multivariable analysis identified S6 origin as the sole independent poor prognostic factor. Jones et al. investigated patients with clinical T1N0 NSCLC (based on the eighth edition of the TNM classification of malignant tumors) undergoing intentional segmentectomy [19]. Their multivariable analysis of disease-free survival and OS revealed that S6 origin was an independent poor prognostic factor for right lower lobe lung cancer. Lu et al. conducted a systematic review of six studies comparing S6 and basal segment lung cancer [2]. Their findings indicated that while no significant difference in prognosis was observed between the two groups when lobectomy or more extensive surgery was performed, S6 lung cancer might have a worse prognosis when segmentectomy was performed for early-stage lung cancer. In summary, S6 lung cancer tends to have a worse prognosis in cohorts including segmentectomy compared to basal segment lung cancer; however, most reports indicate no significant difference in prognosis when lobectomy is performed [2, 3]. Additionally, a meta-analysis by Yu et al*.* demonstrated no significant differences in OS or event-free survival between patients with tumors in the S6 and basal segment. This analysis included six retrospective studies, comprising 730 lobectomies and 70 segmentectomies [20]. The findings of this study, which focused exclusively on pure-solid tumors (known for their oncologic aggressiveness), align with these observations and analyses.

The present study demonstrated no significant difference in the frequency of pathologically confirmed LNM between the two groups. However, superior mediastinal LNM was observed exclusively in the S6 segment group. Previous studies have explored differences in lymphatic metastasis pathways between S6 and basal segment lung cancer [1, 4–8]. Watanabe et al. reported that S6 lung cancer had a direct lymphatic pathway to the upper mediastinum, unlike basal segment lung cancer [4]. Ichinose et al. analyzed LNM patterns based on tumor location and observed that, in bilateral S6 lung cancer, skip N1 metastasis to the interlobar lymph nodes frequently occurred without involvement of the lobar lymph nodes [6]. Handa et al. retrospectively compared 60 cases of S6 lung cancer with 74 cases of basal segment lung cancer [1] and reported lymph node recurrence in four cases (all in the S6 group), with relapses in the superior mediastinal lymph nodes. Yoshimura et al. investigated cN0, solid-dominant NSCLC ≤ 2 cm, comparing 51 cases of S6 lung cancer with 71 cases of basal segment lung cancer. Superior mediastinal LNM was pathologically confirmed in only one case from the S6 group [5]. These reports are consistent with the findings of our study, suggesting that in pure-solid S6 lung cancer, systematic LND or sampling of superior mediastinal lymph nodes may be beneficial for accurate staging.

Our study demonstrated that VPI tended to be more frequent in S6 lung cancer compared to the basal segment. VPI is well known as a poor prognostic factor [21–23]. Previous research on pure-solid NSCLC ≤ 3 cm has also identified VPI as a negative prognostic indicator [24, 25]. Jiwangga et al. retrospectively reviewed patients with recurrent pathological stage I adenocarcinoma following radical resection, categorizing them based on VPI presence [26]. Their multivariable analysis identified VPI as a significant predictor of pleural dissemination as a recurrence pattern after complete resection. Numerous studies have reported unexpectedly detected pleural dissemination at the start of surgery in cases deemed resectable, with incidence rates ranging from 0.9 to 5.3% [27–31]. Furthermore, Li et al. identified VPI as an independent predictor of incidental dissemination identified at surgery initiation [32]. Therefore, when radiological findings suggest VPI, early surgical intervention may improve patient prognosis. As pleural invasion tends to be more common in pure-solid S6 lung cancer, careful clinical management of these cases is vital.

This study has some limitations. First, as a retrospective single-institution analysis with a relatively small sample size and short follow-up period, the findings may not be generalizable to broader patient populations or accurately reflect long-term outcomes. Future multi-institutional studies with larger sample sizes are warranted to validate our findings. Second, a relatively large proportion of FDG-PET data were missing, and programmed death-ligand 1 as well as gene mutation status were not incorporated owing to a significant amount of missing data. Finally, in some patients, the maximum tumor diameter was used as a surrogate when data on pathological invasive size were unavailable. Despite these limitations, this study provides valuable insights for determining treatment strategies for pure-solid S6 lung cancer.

## Conclusion

For pure-solid NSCLC (≤ 5 cm, cN0M0) originating in the lower lobe, lobectomy combined with hilar and mediastinal lymphadenectomy showed no significant prognostic difference between S6 and basal segment lung cancer. However, given that S6 pure-solid NSCLC may exhibit a higher incidence of VPI and distinct lymphatic drainage pathways compared to the basal segment, further investigation is warranted to determine whether segmentectomy can serve as a viable option for radical resection in S6 pure-solid NSCLC.

## Supplementary Information

Below is the link to the electronic supplementary material.

Supplementary Material 1.Supplementary Fig. 1 Survival curves for the two groups among patients with tumors ≤ 2 cm. **a** Overall survival curves for the superior segment (thick line) and basal segment groups (thin line). **b** Recurrence-free survival curves for the superior segment (thick line) and basal segment groups (thin line) (TIFF 4221 KB)

Supplementary Material 2.Supplementary Fig. 2 Survival curves for two groups among patients with tumors > 2 cm. **a** Overall survival curves for the superior segment (thick line) and basal segment groups (thin line). **b** Recurrence-free survival curves for the superior segment (thick line) and basal segment groups (thin line) (TIFF 4015 KB

Supplementary Material 3. (DOCX 22 KB)

## Data Availability

The datasets generated and/or analyzed during the current study are available from the corresponding author on reasonable request.
