# Supplementary figures and images for: Prognostic comparison between superior and basal segments in pure-solid non-small cell lung cancer
Source: Gen Thorac Cardiovasc Surg. 2025 Sep 18;74(3):301–8. doi: 10.1007/s11748-025-02202-6 (PMC12956945; doi:10.1007/s11748-025-02202-6)

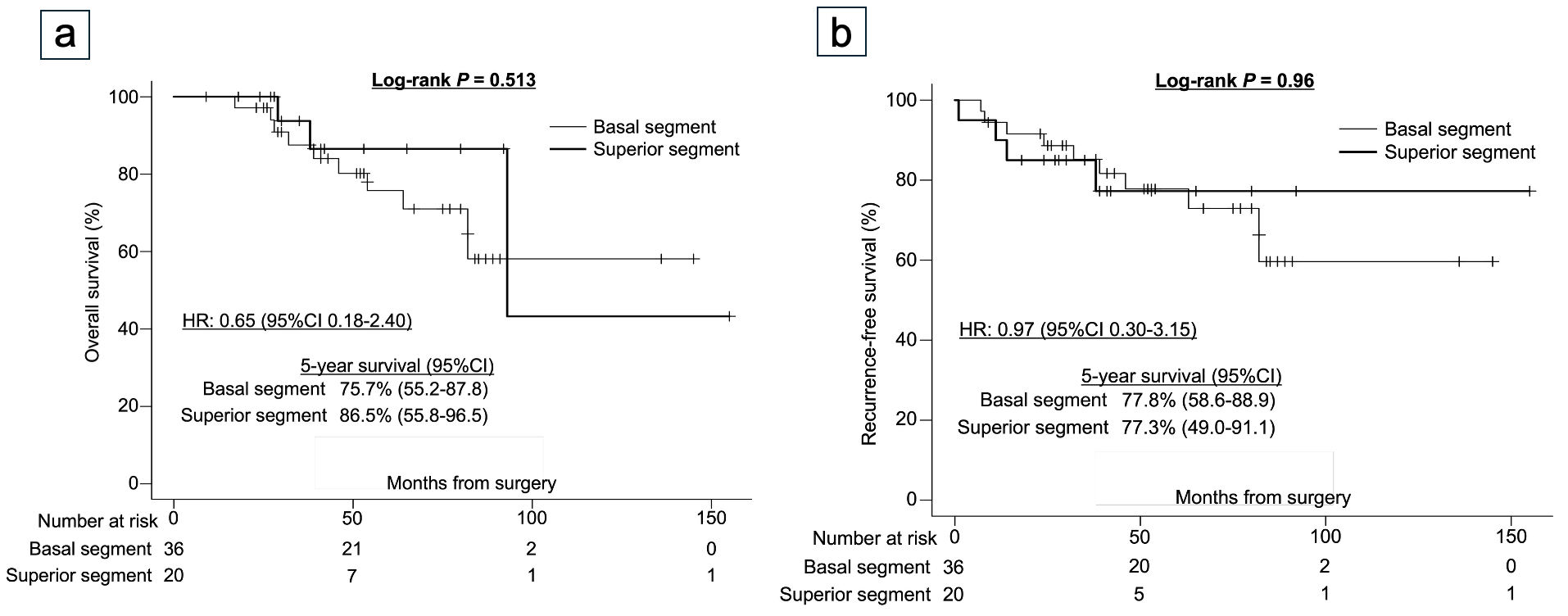

Supplement: Supplementary file 1 — Supplementary Material 1.Supplementary Fig. 1 Survival curves for the two groups among patients with tumors ≤ 2 cm. a Overall survival curves for the superior segment (thick line) and basal segment groups (thin line). b Recurrence-free survival curves for the superior segment (thick line) and basal segment groups (thin line) (TIFF 4221 KB) [file 11748_2025_2202_MOESM1_ESM.tiff]

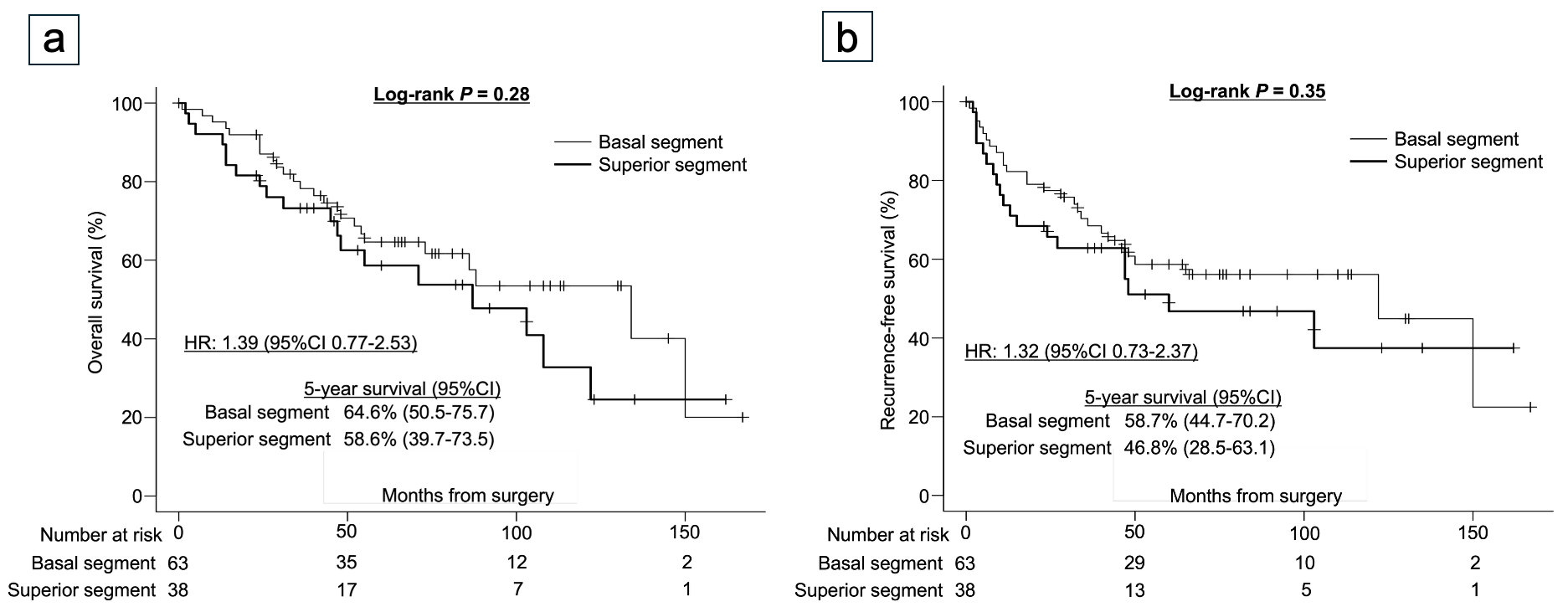

Supplement: Supplementary file 2 — Supplementary Material 2.Supplementary Fig. 2 Survival curves for two groups among patients with tumors > 2 cm. a Overall survival curves for the superior segment (thick line) and basal segment groups (thin line). b Recurrence-free survival curves for the superior segment (thick line) and basal segment groups (thin line) (TIFF 4015 KB [file 11748_2025_2202_MOESM2_ESM.tiff]
