# Supplementary material for: Prognostic comparison between superior and basal segments in pure-solid non-small cell lung cancer
Source: Gen Thorac Cardiovasc Surg. 2025 Sep 18;74(3):301–8. doi: 10.1007/s11748-025-02202-6 (PMC12956945; doi:10.1007/s11748-025-02202-6)
Supplement: Supplementary file 3 — Supplementary Material 3. (DOCX 22 KB) [file 11748_2025_2202_MOESM3_ESM.docx]

**Supplementary Table 1.** Patient characteristics in S6 and basal-segment groups among patients with tumors ≤ 2 cm

| Variables | S6 (N = 20) | Basal segment (N = 36) | *P-*value |
| --- | --- | --- | --- |
| Age (years), mean ± SD | 69.6 ± 8.0 | 67.1 ± 13.3 | 0.44 |
| Sex, n (%) |  |  |  |
| Male | 14 (70.0) | 25 (69.4) | 1.00 |
| Female | 6 (30.0) | 11 (30.6) |  |
| Brinkman Index, mean ± SD | 599 ± 652 | 648 ± 607 | 0.78 |
| %VC (%), mean ± SD | 105 ± 15 | 102 ± 15 | 0.41 |
| FEV1 (mL), mean ± SD | 2326 ± 889 | 2293 ± 562 | 0.87 |
| FEV1% (%), mean ± SD | 71.3 ± 10.5 | 71.3 ± 10.5 | 0.99 |
| Laterality, n (%) |  |  |  |
| Left | 14 (70.0) | 16 (44.4) | 0.12 |
| Right | 6 (30.0) | 20 (55.6) |  |
| Radiological tumor size (mm), mean ± SD | 14.8 ± 2.9 | 15.3 ± 3.5 | 0.53 |
| CEA (ng/mL), median (IQR) | 3.5 (2.6–5.1) | 3.6 (2.6–5.4) | 0.64 |
| SUVmax, median (IQR) | 3.4 (2.2–4.7) | 3.3 (2.0–4.5) | 0.83 |

*CEA,* carcinoembryonic antigen; *FEV1,* forced expiratory volume in one second; *FEV1%,* forced expiratory volume in one second (expressed as forced vital capacity ratio); *IQR,* interquartile range; *%VC,* percentage of predicted vital capacity; *SD,* standard deviation; SUVmax, maximum standardized uptake value; *S6*, superior segment

**Supplementary Table 2.** Pathological and postoperative characteristics in S6 and basal-segment groups among patients with tumors ≤ 2 cm

| Variables | S6 (N = 20) | Basal segment (N = 36) | *P-*value |
| --- | --- | --- | --- |
| Mediastinal LND, n (%) |  |  |  |
| Selective | 29 (80.6) | 20 (100.0) | 0.09 |
| Systematic | 7 (19.4) | 0 (0.0) |  |
| Histology, n (%) |  |  |  |
| Adenocarcinoma | 16 (80.0) | 20 (55.6) | 0.19 |
| Squamous cell carcinoma | 3 (15.0) | 12 (33.3) |  |
| Others | 1 (5.0) | 4 (11.1) |  |
| Pathological N factor, n (%) |  |  |  |
| N0 | 16 (80.0) | 30 (83.3) | 0.90 |
| N1 | 3 (15.0) | 5 (13.9) |  |
| N2 | 1 (5.0) | 1 (2.8) |  |
| Detail of mediastinal LNM, n (%) |  |  |  |
| Superior | 0 (0.0) | 0 (0.0) | NA |
| Inferior | 1 (5.0) | 1 (2.8) | 1.00 |
| Pathological stage, n (%) |  |  |  |
| IA1 | 3 (15.0) | 7 (19.4) | 0.61 |
| IA2 | 8 (40.0) | 10 (27.8) |  |
| IA3 | 2 (10.0) | 9 (25.0) |  |
| IB | 3 (15.0) | 3 (8.3) |  |
| IIA | 0 (0.0) | 1 (2.8) |  |
| IIB | 2 (10.0) | 5 (13.9) |  |
| IIIA | 1 (5.0) | 1 (2.8) |  |
| IIIB | 1 (5.0) | 0 (0.0) |  |
| IVA | 0 (0.0) | 0 (0.0) |  |
| Lymphatic invasion, n (%) | 6 (30.0) | 10 (27.8) | 1.00 |
| Venous invasion, n (%) | 11 (55.0) | 13 (36.1) | 0.28 |
| Visceral pleural invasion, n (%) | 5 (25.0) | 6 (16.7) | 0.69 |
| Pulmonary metastasis, N (%) | 1 (5.0) | 0 (0.0) | 0.76 |
| Adjuvant therapy, N (%) | 4 (21.1) | 3 (8.3) | 0.36 |

*S6*, superior segment; *LND,* lymph node dissection; *LNM,* lymph node metastasis; *NA*, non-applicable

**Supplementary Table 3.** Patient characteristics in S6 and basal-segment groups among patients with tumors > 2 cm

| Variables | S6 (N = 38) | Basal segment (N = 63) | *P-*value |
| --- | --- | --- | --- |
| Age (years), mean ± SD | 73.1 ± 8.6 | 72.0 ± 8.4 | 0.53 |
| Sex, n (%) |  |  |  |
| Male | 26 (68.4) | 42 (66.7) | 1.00 |
| Female | 12 (31.6) | 21 (33.3) |  |
| Brinkman Index, mean ± SD | 811 ± 705 | 770 ± 735 | 0.78 |
| %VC (%), mean ± SD | 103 ± 17 | 103 ± 16 | 1.00 |
| FEV1 (mL), mean ± SD | 2137 ± 596 | 2134 ± 690 | 0.98 |
| FEV1% (%), mean ± SD | 70.1 ± 9.7 | 71.3 ± 9.5 | 0.56 |
| Laterality, n (%) |  |  |  |
| Left | 15 (39.5) | 32 (50.8) | 0.37 |
| Right | 23 (60.5) | 31 (49.2) |  |
| Radiological tumor size (mm), mean ± SD | 33.0 ± 7.9 | 30.9 ± 8.0 | 0.21 |
| CEA (ng/mL), median (IQR) | 4.4 (2.7–5.8) | 5.9 (4.0–8.8) | 0.03 |
| SUVmax, median (IQR) | 8.7 (4.0–12.4) | 6.0 (3.0–8.6) | 0.009 |

*CEA,* carcinoembryonic antigen; *FEV1,* forced expiratory volume in one second; *FEV1%,* forced expiratory volume in one second (expressed as forced vital capacity ratio); *IQR,* interquartile range; *%VC,* percentage of predicted vital capacity; *SD,* standard deviation; SUVmax, maximum standardized uptake value; *S6*, superior segment

**Supplementary Table 4.** Pathological and postoperative characteristics in S6 and basal-segment groups among patients with tumors > 2 cm

| Variables | S6 (N = 38) | Basal segment (N = 63) | *P-*value |
| --- | --- | --- | --- |
| Mediastinal LND, n (%) |  |  |  |
| Selective | 22 (57.9) | 41 (65.1) | 0.61 |
| Systematic | 16 (42.1) | 22 (34.9) |  |
| Histology, n (%) |  |  |  |
| Adenocarcinoma | 21 (55.3) | 44 (69.8) | 0.33 |
| Squamous cell carcinoma | 13 (34.2) | 15 (23.8) |  |
| Others | 4 (10.5) | 4 (6.3) |  |
| Pathological N factor, n (%) |  |  |  |
| N0 | 30 (78.9) | 46 (73.0) | 0.51 |
| N1 | 3 (7.9) | 10 (15.9) |  |
| N2 | 5 (13.2) | 7 (11.1) |  |
| Detail of mediastinal LNM, n (%) |  |  |  |
| Superior | 2 (5.3) | 0 (0.0) | 0.27 |
| Inferior | 4 (10.5) | 7 (11.1) | 1.00 |
| Pathological stage, n (%) |  |  |  |
| IA1 | 0 (0.0) | 0 (0.0) | 0.32 |
| IA2 | 1 (2.6) | 5 (7.9) |  |
| IA3 | 5 (13.2) | 9 (14.3) |  |
| IB | 17 (44.7) | 21 (33.3) |  |
| IIA | 0 (0.0) | 7 (11.1) |  |
| IIB | 7 (18.4) | 8 (12.7) |  |
| IIIA | 7 (18.4) | 9 (14.3) |  |
| IIIB | 1 (2.6) | 2 (3.2) |  |
| IVA | 0 (0.0) | 2 (3.2) |  |
| Lymphatic invasion, n (%) | 12 (31.6) | 24 (38.1) | 0.65 |
| Venous invasion, n (%) | 24 (63.2) | 33 (52.4) | 0.40 |
| Visceral pleural invasion, n (%) | 23 (60.5) | 22 (34.9) | 0.02 |
| Pulmonary metastasis, N (%) | 4 (10.5) | 3 (4.8) | 0.48 |
| Adjuvant therapy, N (%) | 11 (28.9) | 12 (19.0) | 0.37 |

*S6*, superior segment; *LND,* lymph node dissection; *LNM,* lymph node metastasis
